# The Intention of Delivery Room Staff to Encourage the Presence of Husbands/Partners at Cesarean Sections

**DOI:** 10.1155/2011/192649

**Published:** 2011-06-07

**Authors:** Yaira Gutman, Nili Tabak

**Affiliations:** ^1^Assaf Harofeh Hospital, 70300 Zerifin, Israel; ^2^M.A. Program, Department of Nursing, Tel Aviv University, 69978 Tel Aviv, Israel; ^3^Nursing Department, School of Health Professions, Sackler Faculty of Medicine, Tel Aviv University, 69978 Tel Aviv, Israel

## Abstract

*Objective*. In recent years, more and more delivery rooms have allowed husbands/partners to be present during a Cesarean section Nonetheless, many still oppose the idea. The study is designed to investigate the attitudes of Israeli gynecologists, anesthetists, operating-room nurses, and midwives on this issue. *Design*. The study's theoretical model comes from Fishbein and Ajzen's theory of reasoned action. A self-administered questionnaire was submitted to convenience sample. *Subjects*. 96 gynecologists, anesthetists, midwives, and operating-room nurses. *Results*. Significant differences were found between the occupational subgroups. Most of the findings supported the four hypotheses tested and confirmed earlier studies designed to verify the theoretical model. *Conclusions*. The main conclusion drawn is that delivery and operating-room staff need to be trained in the skills needed to promote the active participation of the baby's father in delivery and, if necessary, in a Cesarean section.

## 1. Introduction

In recent years, as Cesarean sections themselves have become more common, more and more delivery rooms have allowed husbands/partners to be present during a Cesarean section, arguing that this reduces the mother's anxiety, cements the father's role in childrearing, and reflects current occupational positions on patient autonomy of decision and quality of care [1–3].

Indeed, the husband/partner's right to be present has been extended to any close relative or husband the birthing mother wants alongside her. Nonetheless, many still oppose the idea, arguing that the “invited guest” often finds the experience emotionally painful, even traumatic, that they can interfere with the staff's work and decision making, and that their close attendance might even multiply already frequent enough malpractice claims [4–6].

Apart from this legal consideration, the attitudes of doctors and nurses on this issue have been found to be influenced by their occupational training and ethos, their cultural background, and their previous experience of admitting husbands to an operating room [7]. Nurses have reported that they felt uncomfortable and uneasy at having family members watching their every move [4, 7].

The researchers have found very little research data on the above issues from Israel, and one of this study's aims is to fill this gap for Israel and break new ground. The study is designed to investigate the attitudes of doctors and nurses in operating rooms and delivery rooms to the presence of the husband during a Cesarean section and the association between these attitudes and the staffers' willingness to promote this organizational change. The study's ultimate goal is to improve the quality of care of the birthing mother and her family before and during a Cesarean section.

## 2. The Theoretical Model

**Figure 1:**
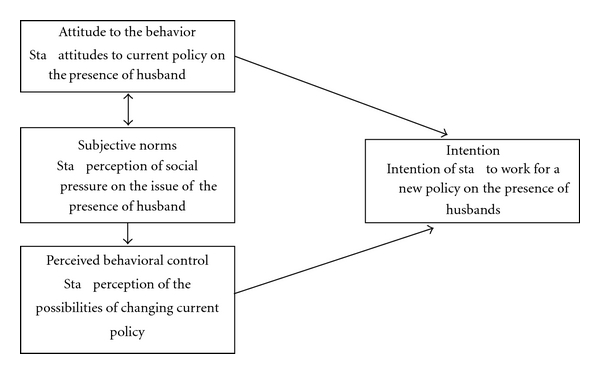
The Study's theoretical model derived from the Ajzen and Madden theory of reasoned action.

The study's theoretical model comes from Fishbein and Ajzen's theory of reasoned action (Figure 1) [8]. The theory aims to furnish a means of understanding and predicting most of human behavior by means of a small number of concepts—intentions, attitude, beliefs, perceived social pressure, and perceived behavioral control (PBC). Fishbein and Ajzen, while appreciating that social reality is highly complex, nevertheless offer to explain it with a relatively simple theory, and their empirically testable theory offers a systematic means of investigating behavior across a wide range of contexts. Applying it to different populations, it can identify the factors distinguishing groups from each other and explain why they behave differently in the same situation. Since the present study aims to predict the behavior of four occupational groups (gynecologists, anesthetists, operating-room nurses, and midwives) who differ both by current occupation and past training, the Fishbein and Ajzen theory seems a suitable tool. The theory's authors claim that behavioral intention is the product of two independent variables—a person's attitudes to the behavior in question and their perception of social norms (subjective norms).

(a) *A person's attitudes to the behavior in question* are defined as the sum total of their beliefs about the behavior.

(b) *Subjective norms* are defined as how the person perceives the interpersonal and social repercussions of their behaving in the given manner.

In 1986, Ajzen and Madden expanded their theory of reasoned action by adding the construct of perceived behavioral control (PBC) [9]. This is defined as a person's confidence in their ability to carry through a given behavior. Do they have the personal resources necessary and will their working environment allow them to carry the behavior through? The more they believe they have both, the stronger their PBC will be.

From the Ajzen and Madden theory, we derive the basic premise of this study—that there is a direct relation between, on the one hand, operating and delivery room staff attitudes to the presence of a husband during Cesarean sections, their perceived behavioral control, and their perception of social pressure on this issue and, on the other hand, their behavioral intention to act to bring about this change.

The more favorable the staffers' own attitudes, the stronger their perceived behavioral control, and the more supportive they perceive their working environment to be on the issue of allowing a husband's presence, the stronger will be their intention to promote this change.

## 3. Research Hypotheses

(1) There is a positive relation between staffer attitudes (gynecologists, anesthetists, midwives, and operating-room nurses) to admitting a husband to Cesarean sections, their subjective norms and their perceived behavioral control on the prospect of bringing about this behavior.

(2)The four occupational subgroups will differ on their attitudes and perceived behavioral control.

(3) There is a positive relation between staffer attitudes and their behavioral intention to admit husbands during a Cesarean section.

(4) There is a positive relation between staff's perceived behavioral control and their intention to admit husbands during a Cesarean section.

## 4. Methods

### 4.1. The Sample

The sample was a convenience sample composed of gynecologists, anesthetists, midwives, and operating-room nurses at the Assaf Harofeh hospital in Tel Aviv. It comprised all the staffers working in two obstetrics departments, a gynecology department, a high-risk pregnancy department, and 12 delivery rooms. Of the 96-strong sample, 51% were nurses, almost equally divided between operating-room nurses and midwives, and 49% were doctors (45% anesthetists and 55% gynecologists). Of the 49 nurses, 31 had an academic nursing qualification (B.A. or M.A.), and 20 had graduated a regular basic training program. Almost all had taken some form of advanced training. Of the 47 doctors, 19 were interns, and 28 were qualified specialists.

Two-thirds of the sample, doctors and nurses, were women. Seniority ranged from 1–37 years and age from 26–60.

### 4.2. The Research Instrument

The single instrument used was a self-administered questionnaire, constructed and validated by the senior researcher and identical for all participants. It was designed to detect associations between the five variables it measured and whether they affected the components of the research model. Not finding a validated and reliable instrument in the published literature, the senior researcher based the questionnaire, as noted, on the expanded Ajzen and Madden theory of reasoned action, supplementing it with questions taken from three other researchers who had used this theory to investigate behavior in the context of various health care issues (nurses' promotion of patient privacy, emergency nurses' attitudes to allowing family presence during invasive and resuscitation procedures, and the public's willingness to donate organs for transplant).

The first draft of the questionnaire was reviewed by four medical experts from the fields of gynecology, anesthesiology, nursing, and statistics and then pilot-tested on eight subjects, two each from the four selected professions (gynecologists, anesthetists, midwives, and operating-room nurses). It was corrected and revised in light of their comments. The Cronbach Alpha scores for the internal reliability of Sections 2–5 of the questionnaire were all high over 0.8.

**Section 1**

16 questions on participants' sociodemographic variables.

**Section 2**

Staff behavioral attitudes to admitting a husband to Cesarean sections.

**Section 3**

Perceived behavioral control on admitting husbands to Cesarean sections. A subject's self-confidence on this issue stems from internal and external factors: internal factors: confidence in his/her own ability and knowledge to carry through the behavior and external factors: fear of legal suits and the suitability of operating room conditions for husbands to be in attendance.

**Section 4**

The subject's behavioral intention to admit husbands to Cesarean sections. Will he/she find legitimate reasons to prevent the husband's admission or, instead, find solutions to problems that may arise?

**Section 5**

The subject's perception of social norms in their working environment on admitting a husband to Cesarean sections and the resultant pressure on him/her.

All questions were answered on a 6-point Likert scale from “never” to “always” and the mean scores were used to represent the range of individual scores. Data were analyzed using Pearson correlation coefficients and ANOVA.

### 4.3. Research Process

The questionnaire was distributed to staff shortly after the operating rooms had begun allowing a husband to attend Cesarean sections. After the management of the Assaf Harofeh hospital and its Helsinki committee had given permission for the research study the senior researcher distributed the questionnaires to all the staffers registered as working in the four selected units, who returned them later completed.

## 5. Results

**Hypothesis 1**

There is a positive relation between *staffer attitudes* (gynecologists, anesthetists, midwives, and operating-room nurses) to admitting a husband to Cesarean sections, their *subjective norms*, and their *perceived behavioral control *on the prospect of bringing about this behavior.

Table 1 shows that for both occupational groups (doctors and nurses) and all four subgroups (gynecologists, anesthetists, midwives and operating-room nurses), there is a strong positive correlation (*P* < .001) between their attitudes and their PBC. The Pearson correlation is highest for the midwives (*R* = 0.749) and lowest for the gynecologists (*R* = 0.506). Although the significance level is lower for the gynecologists than for the other groups (*P* < .006) it is still high.

The correlation between subjective norms and PBC is also strong and statistically significant for the whole sample, but only for three of the four subgroups (not for midwives). Hypothesis 1 can, therefore, be considered partially confirmed.

**Hypothesis 2**

The four occupational subgroups will differ on their *attitudes* and *perceived behavioral control*.

Nurses (mean = 3.49; SD = 0.05) were somewhat more supportive of admitting a husband to Cesarean sections than doctors (mean = 3.24; SD = 0.55). (On subjective norms, no significant between-group differences were found.) The nurses' perceived behavioral control (mean = 2.95; SD = 0.31) was also significantly stronger than that of the doctors (mean = 2.72; SD = 0.38), and there were also significant differences between the subgroups.

Hypothesis 2 was confirmed.

**Hypothesis 3**

There is a positive relation between staffer *attitudes*, *subjective norms*, and their *behavioral intention* to admit a husband during a Cesarean section.

Table 2 shows that for both occupational groups (doctors and nurses) (*P* < .001) and all four subgroups (*P* < .05) there is a strong positive correlation between their attitudes and their behavioral intention. The Pearson correlation is highest for the anesthetists (*R* = 0.674) and lowest for the gynecologists (*R* = 0.378). The correlation between subjective norms and behavioral intention is strong and statistically significant for both occupational groups and all four subgroups, this time with the gynecologists displaying the strongest Pearson correlation coefficient.

Hypothesis 3 can, therefore, be considered confirmed.

**Hypothesis 4**

There is a positive relation between staff's *perceived behavioral control* and their *behavioral intention* to admit a husband during a Cesarean section.

Table 3 shows a strong positive correlation between staff's perceived behavioral control and their behavioral intention to admit a husband during a Cesarean section, for both occupational groups and three subgroups, ranging from *R* = 0.355 to *R* = 0.632, with the anesthetists the exception in not displaying a statistically significant correlation.

Hypothesis 4 can be considered partially confirmed.

## 6. Discussion and Clinical Implications

The aims of this study were to see if there is an association between attitudes and beliefs of the doctors and nurses involved in delivering babies as regards the policy of admitting a patient's “husband” to Cesarean sections and their intended behavior on that issue. The researchers also wanted to know if there were differences on this issue between the occupational subgroups.

Most of the findings supported the hypotheses derived from the Ajzen and Fishbein [8] and Ajzen and Madden [9] theory of reasoned action and confirmed earlier studies designed to verify this theory.

Hypothesis 1 was partially confirmed; that is, staffer attitudes to admitting a husband to Cesarean sections were found to be positively associated with their PBC on this issue. The correlation between subjective norms and PBC is also strong for three of the four subgroups. Midwives are the exception. It is pertinent to note here that whereas the three other occupational groups work regularly together in the operating room, the brunt of midwives' work is in pre-natal preparation. In the normal course of a pregnancy, the mother is prepared and instructed by a midwife, up to and including events in the delivery room itself. Only when this “normal course” breaks down, and it is decided to deliver by Cesarean, is the mother taken over by the operating room team. Midwives are strongly supportive of the husband's close involvement in pregnancy and birth, including in a Cesarean delivery, and in this, they are responding to their clients' pressure, but the operating room staff has the veto prerogative. It is this inability to obtain their clients' wishes that explains the nonstatistically significant correlation between the midwives' subjective norms and their PBC.

Hypothesis 2, that the four occupational subgroups will differ on their attitudes and perceived behavioral control, was confirmed. Kotkis [10] also found disparities between occupational groups on the issue of allowing family members' presence during resuscitation and other invasive procedures.

Hypothesis 3: In other words, staffer attitudes to admitting a husband to Cesarean sections are positively associated with their behavioral intention on this issue. Analysis of variance found that attitudes were a significant predictor of behavioral intention. In other words, if we want to modify intentions and behavior, we have to impact on attitudes and to change the beliefs which underlie attitudes we have to tie them into positive practical outcomes. It follows that the key to change lies in education and training.

In the Ajzen and Madden theory, attitude is a product of beliefs, which in turn represent the individual's knowledge on a given issue. Supplying new information can, therefore, alter beliefs and attitudes, and this is what indeed happened in Bassler's study of intensive care nurses [11].

Beliefs and “knowledge” concerning a given behavior are also the outcome of the believer's previous experience with that behavior so that supplying obstetricians and operating room nurses with new information about the consequences of admitting a husband to Cesarean sections could have some positive effect if perhaps an effect limited by their own previous experience with the behavior.

Hypothesis 4: the hypothesized positive relation between staff's perceived behavioral control and their behavioral intention was confirmed for all subgroups except for the anesthetists.

The most probable explanation for anesthetists being an exception to the rule is not that the anesthetists score low on PBC but that they have no intention of working to admit husbands to Cesarean sections. Sakala's survey of nurses, midwives, gynecologists, and anesthetist's attitudes to admitting fathers to Cesarean sections found that the anesthetists were the most skeptical about the idea of the four groups [12]. Anesthetists provide services to gynecology departments but are not involved personally with the birthing mothers and their partners. They see them for the first time in the operating room, whereas the other staff groups may have known the couple throughout the pregnancy. An anesthetist's work is extremely intense and critical, and this too may cause them not to welcome “guests”, all the more so as the place assigned to these guests in an operating room is the area which the anesthetist is responsible for.

## 7. Conclusions

(i) The chief predictor of PBC and behavioral intention is a staffer's behavioral attitudes.

(ii) Behavioral intention is a function of behavioral attitudes and occupation as Table 2 shows.

(iii) The way to improve the behavioral intention to admit a husband to Cesarean sections is by altering individual attitudes and environmental norms on the issue. Therefore, staff training should be altered to include material designed to raise awareness of the advantages of admitting a husband to Cesarean sections and to show the practice in a positive light.

(iv) Topics to be addressed in by this material should include reducing staff anxiety, infection prevention, and legal issues.

(v) Working groups need to be set up on improving the physical conditions in delivery and operating rooms to make them fit to accommodate a husband.

(vi) Delivery and operating room staff need to be trained in workshops and/or training courses in the skills needed to promote the active participation of the baby's mother and father in the delivery and, if necessary, in the Cesarean section.

(vii) performance protocols should be drawn up to guide staff on realizing the new policy.

(viii) Adding staff to the current complement in delivery and operating rooms would reduce the pressure added by the new policy and give staff the confidence that they have the resources to carry through the change of policy.

## Figures and Tables

**Table 1:** Pearson correlation coefficients between staff attitudes, subjective norms, and perceived behavioral control.

		Total sample	Doctors v. nurses		4 Occupational subgroups			
			Doctors	Nurses	Gynecologists	Anesthetists	Delivery room nurses	Midwives
	*N*	96	47	49	26	21	26	23

Staff attitudes	Coef. *R* (*P*)	0.620 (.000)	0.583 (.000)	0.602 (.000)	0.506 (.000)	0.667 (.000)	0.613 (.000)	0.749 (.000)

Subjective norms	Coef. *R* (*P*)	0.404 (.000)	0.492 (.000)	0.344 (.000)	0.703 (.000)	0.469 (.002)	0.464 (.000)	0.213 (.177)

**Table 2:** Pearson correlation coefficients between staff attitudes, subjective norms and behavioral intention.

		Total sample	Doctors v. nurses		4 Occupational subgroups			
			Doctors	Nurses	Gynecologists	Anesthetists	Delivery room nurses	Midwives
	*N*	96	47	49	26	21	26	23

Staff attitudes	Coef. *R* (*P*)	0.639 (.000)	0.555 (.000)	0.666 (.000)	0.378 (.034)	0.674 (.001)	0.617 (.001)	0.629 (.001)

Subjective norms	Coef. *R* (*P*)	0.517 (.000)	0.598 (.000)	0.481 (.000)	0.741 (.000)	0.557 (.007)	0.586 (.001)	0.369 (.05)

**Table 3:** Pearson correlation coefficients between behavioral intention and perceived behavioral control.

Total sample		Doctors v. nurses		4 Occupational subgroups			
		Doctors	Nurses	Gynecologists	Anesthetists	Delivery room nurses	Midwives
*N*	96	47	49	26	21	26	23

Coef. *R* (*P*)	0.536 (.000)	0.437 (.000)	0.559 (.000)	0.541 (0.003)	0.355 (.068)	0.632 (.000)	0.586 (.003)
